# Beneath the Surface: A Rash That Uncovered Renal Autoimmunity

**DOI:** 10.7759/cureus.92521

**Published:** 2025-09-17

**Authors:** Matheus Vasconcelos Goes Mendes, John R Dahl

**Affiliations:** 1 Unidade de Terapia Intensiva (UTI 4), Hospital Português, Salvador, BRA; 2 Department of Internal Medicine, University of South Florida, Tampa, USA

**Keywords:** acute kidney injury, antinuclear antibody, generalized rash, lupus nephritis, systemic lupus erythema

## Abstract

We report the case of a 42-year-old male who presented with a generalized rash, acute kidney injury, anemia, and positive antinuclear antibodies (ANA). Serologic testing revealed elevated anti-double-stranded DNA (dsDNA) levels and hypocomplementemia, raising concern for systemic lupus erythematosus (SLE). Kidney biopsy confirmed Class III (focal proliferative) lupus nephritis according to the International Society of Nephrology/Renal Pathology Society 2003 classification. Immunofluorescence demonstrated full-house staining (positive for IgG, C3, C1q, and light chains) with weaker signals for IgA and IgM. Electron microscopy revealed subepithelial and mesangial electron-dense deposits. Early recognition and timely initiation of immunosuppressive therapy led to clinical improvement and stabilization of renal function.

## Introduction

Systemic lupus erythematosus (SLE) is a chronic autoimmune disease with multisystem involvement and highly variable clinical manifestations. It most commonly affects women of childbearing age, but can occur in individuals of any age, sex, or ethnicity. SLE in men is rare, with a female-to-male ratio of approximately 9:1, and often presents more severely. Male patients are more likely to exhibit prominent skin and neurologic involvement, cytopenias, thrombosis, vasculitis, renal disease, and serositis [1].

The pathophysiology of SLE involves loss of immune tolerance, production of autoantibodies - particularly antinuclear antibodies (ANA) - and immune complex deposition. These processes result in widespread tissue inflammation and organ damage [1-3].

Renal involvement, or lupus nephritis (LN), is among the most serious and potentially life-threatening manifestations of SLE. It is estimated to occur in approximately 40-60% of adults with lupus and up to 80% of children with the disease [4]. LN often presents within the first few years of diagnosis but also may occasionally be the initial manifestation, even in the absence of other classical signs or symptoms of lupus.

Clinically, the disease may range from asymptomatic proteinuria or microscopic hematuria to overt nephritic or nephrotic syndromes, or rapidly progressive glomerulonephritis leading to acute kidney injury (AKI) [5]. Because its presentation can mimic other renal diseases, especially in the setting of systemic symptoms such as rash, anemia, or fatigue, the diagnosis requires a high index of suspicion.

A kidney biopsy is the gold standard for the diagnosis and classification of LN. The International Society of Nephrology/Renal Pathology Society (ISN/RPS) classification system categorizes LN into six classes based on histopathological findings, with proliferative forms (Class III and IV) associated with the worst renal outcomes if not treated aggressively [6].

Despite advances in immunosuppressive therapies over the years, lupus nephritis remains a major cause for morbidity and mortality in patients with SLE. Delayed recognition and treatment significantly increase the risk of progression to end-stage renal disease (ESRD) [7].

This case illustrates the diagnostic challenge posed by lupus nephritis in a patient with unexplained rash, renal dysfunction, and hematologic abnormalities.

## Case presentation

A 42-year-old Hispanic male with no known past medical history presented to the emergency department with complaints of progressive arthralgia, photosensitive rash, bilateral lower extremity edema and decreased urine output over the preceding three weeks. He denied fever, weight loss, hematuria, recent infections or recent travels. There was a positive family history for SLE (aunt, uncle and cousin).

**Figure 1:**
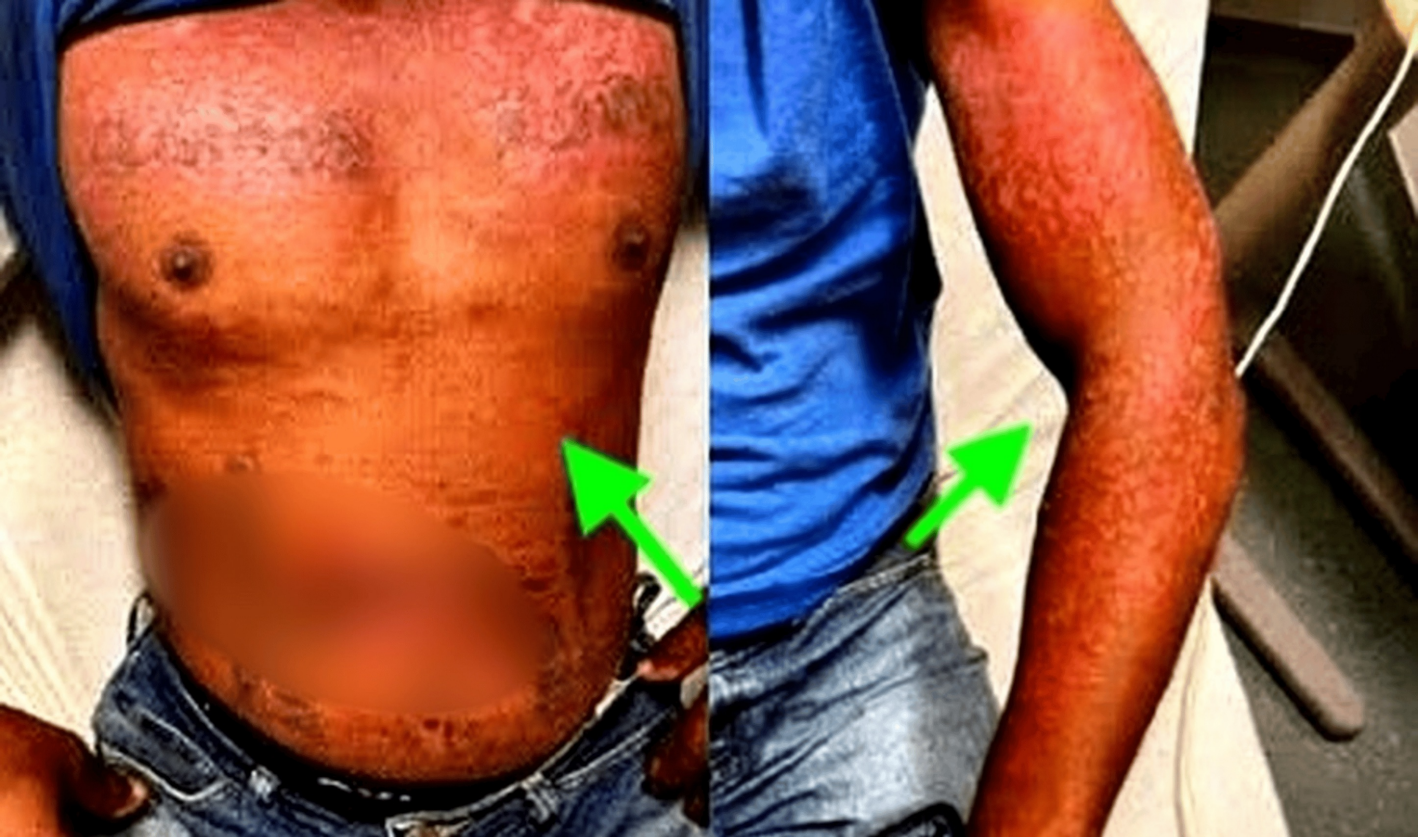
Upper body rash (green arrows).

On examination, dermatologic inspection revealed erythematous patches over sun-exposed areas including the cheeks and upper extremities, consistent with a photosensitive rash (Figure 1). No oral ulcers, alopecia, synovitis, or neurologic deficits were noted. There was trace edema in lower extremities and thigh area. Hypertension was also present during admission.

Initial laboratory workup revealed acute kidney injury with a serum creatinine of 1.90 mg/dL (baseline 1.2 per laboratory 2023), blood urea nitrogen (BUN) of 50mg/dL, and a mildly low hemoglobin level of 10.9 g/dL (Table 1). Urinalysis upon admission showed proteinuria but no hematuria. Additional labs were notable for low complement levels, positive ANA and positive anti-double-stranded DNA (dsDNA) antibodies.

**Table 1:** Laboratory results. BUN: blood urea nitrogen, ANA: antinuclear antibody

Parameter	Result	Reference Range
WBC	3.45 x10³/µL	4.5-11.0 x10³/µL
Hemoglobin	10.9 g/dL	13.5-17.5 g/dL
Creatinine	1.90 mg/dL	0.7-1.3 mg/dL
BUN	50.0 mg/dL	8.0-24.0 mg/dL
ANA	Positive	Negative
Complement (C3/C4)	Low	C3: 88-201 mg/dL; C4: 15-45mg/dL
Urinalysis	Proteinuria; no hematuria	No proteinuria or hematuria

Given the combination of rash, anemia, proteinuria, positive ANA, and hypocomplementemia, systemic lupus erythematosus with renal involvement was suspected. The patient was started on intravenous steroid for three days, followed by high-dose oral prednisone.

**Figure 2:**
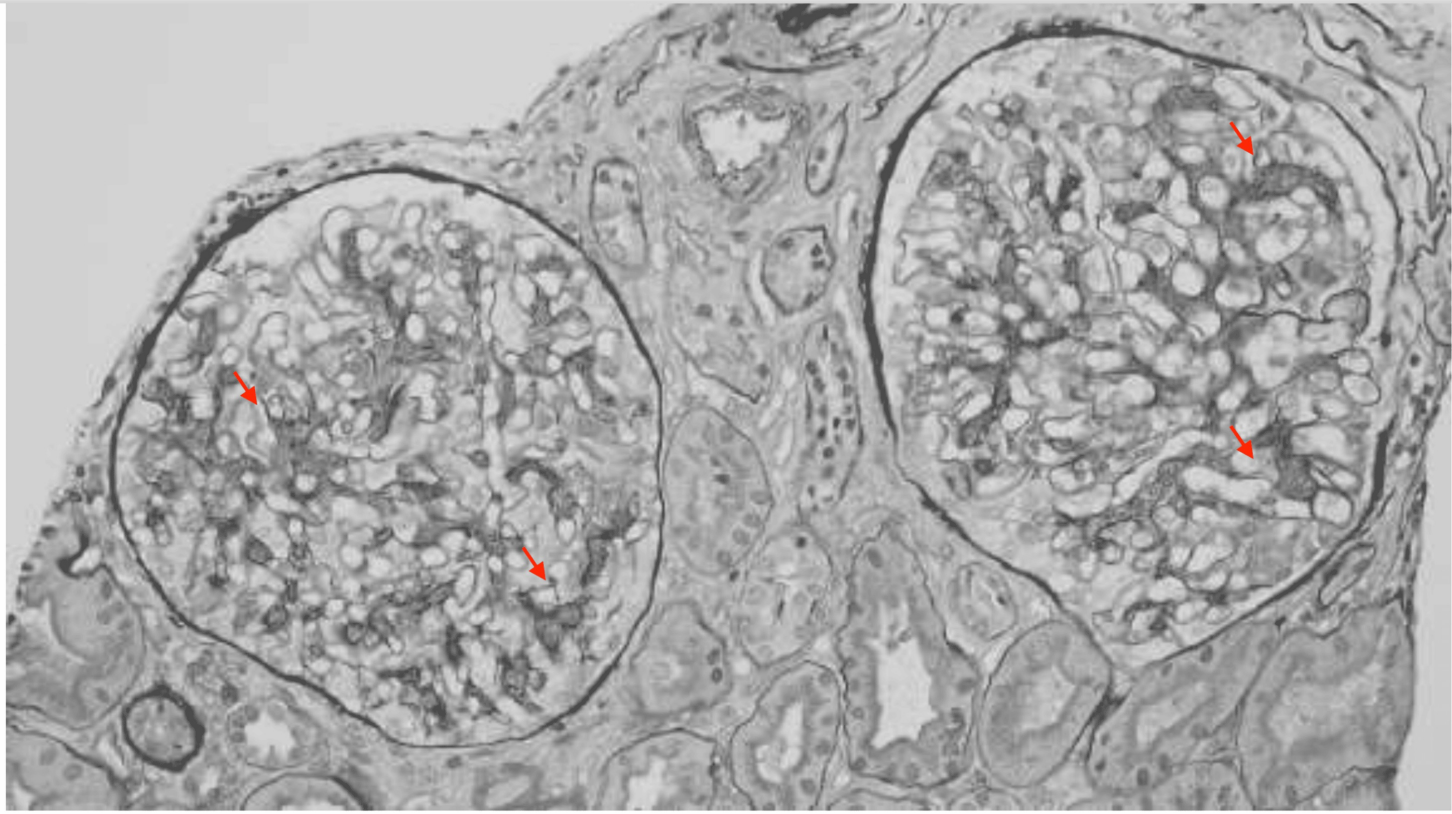
Periodic Acid-Schiff (PAS) and silver stains show segmental endocapillary hypercellularity a small subset of glomeruli (red arrows).

**Figure 3:**
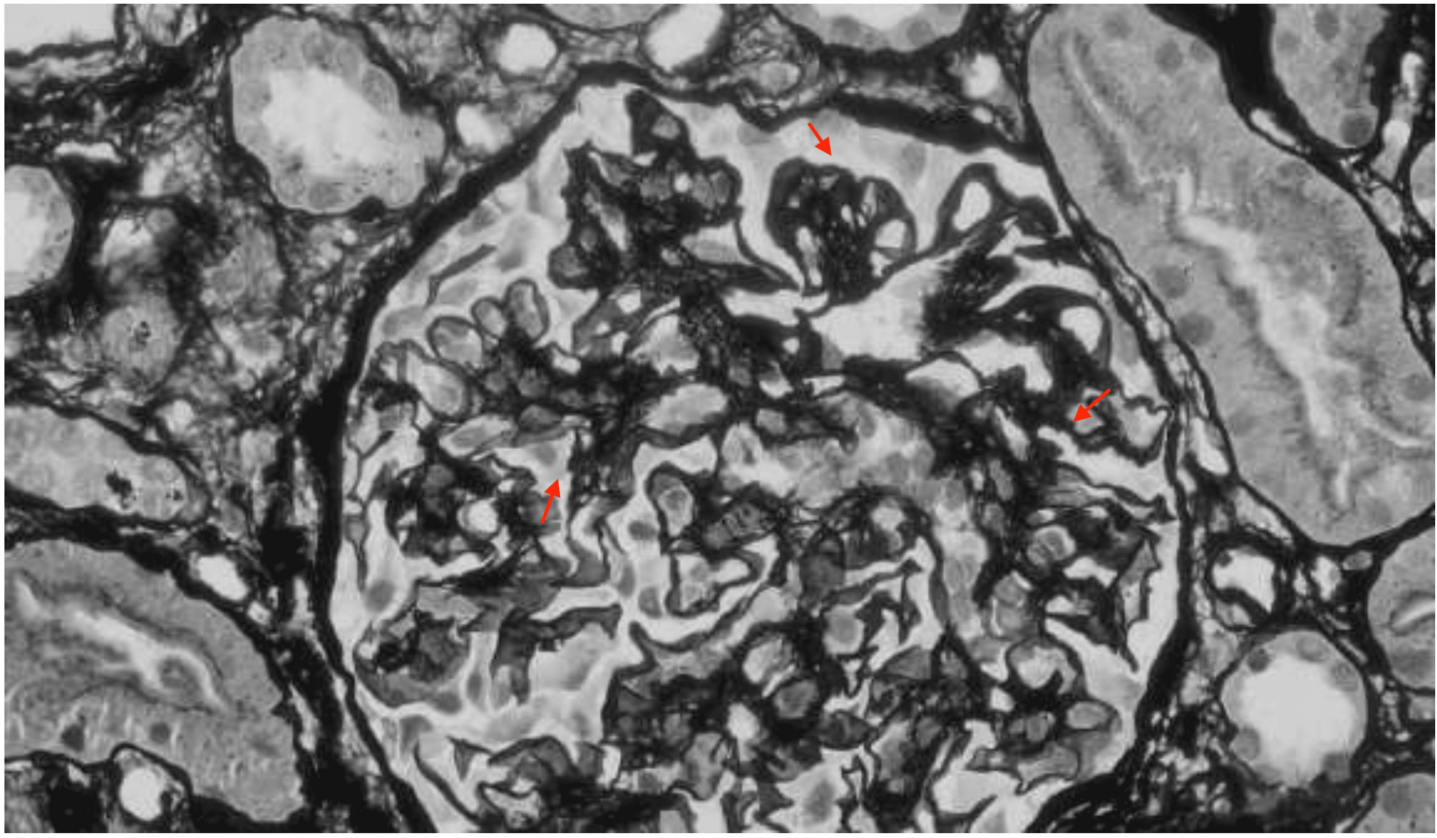
Periodic Acid-Schiff (PAS) and silver stains show segmental endocapillary hypercellularity a small subset of glomeruli (red arrows).

**Figure 4:**
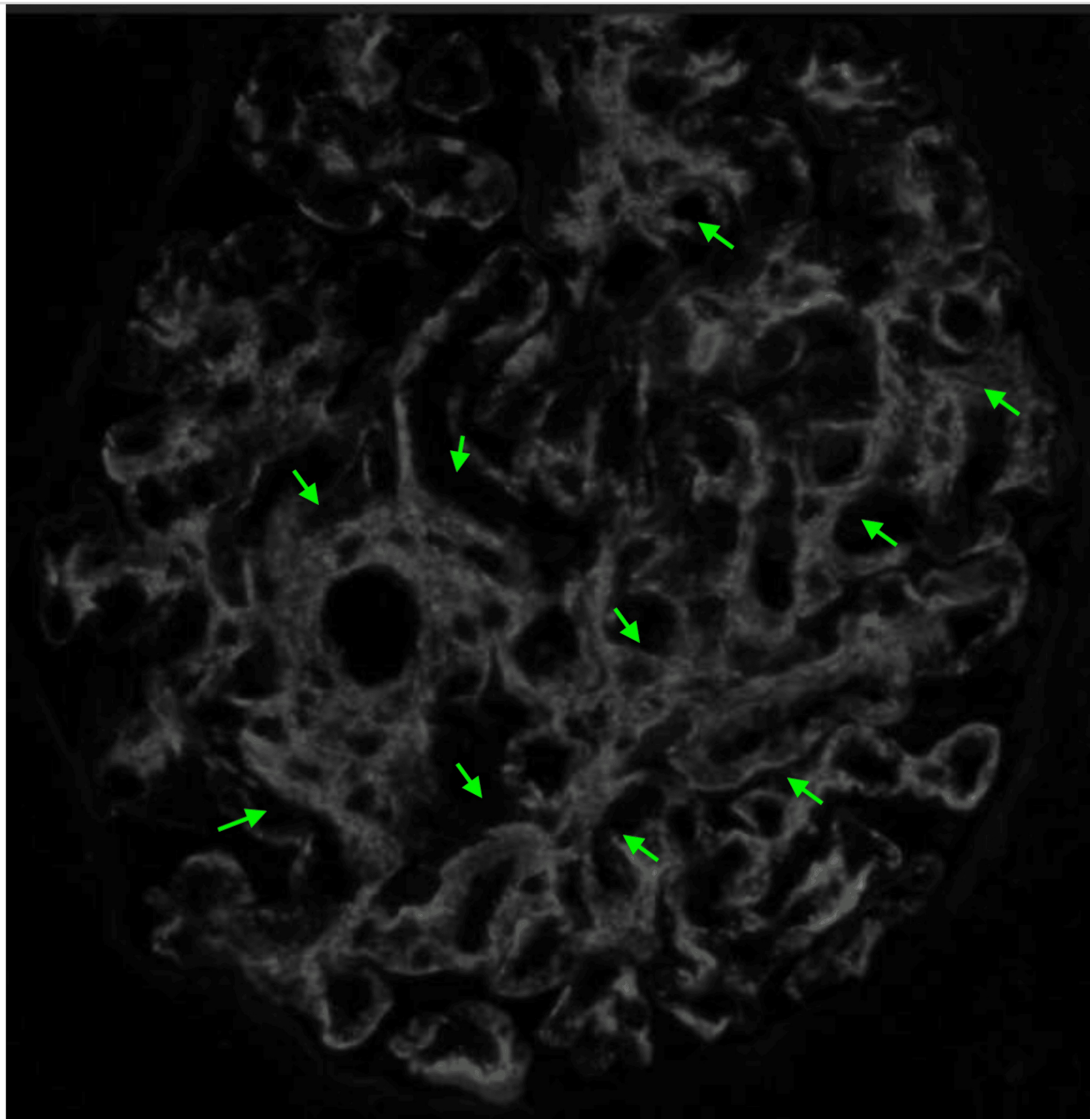
Immunofluorescence (IF) shows granular stain pattern primarily in mesangium with extension to capillary walls; IgG (green arrows).

**Figure 5:**
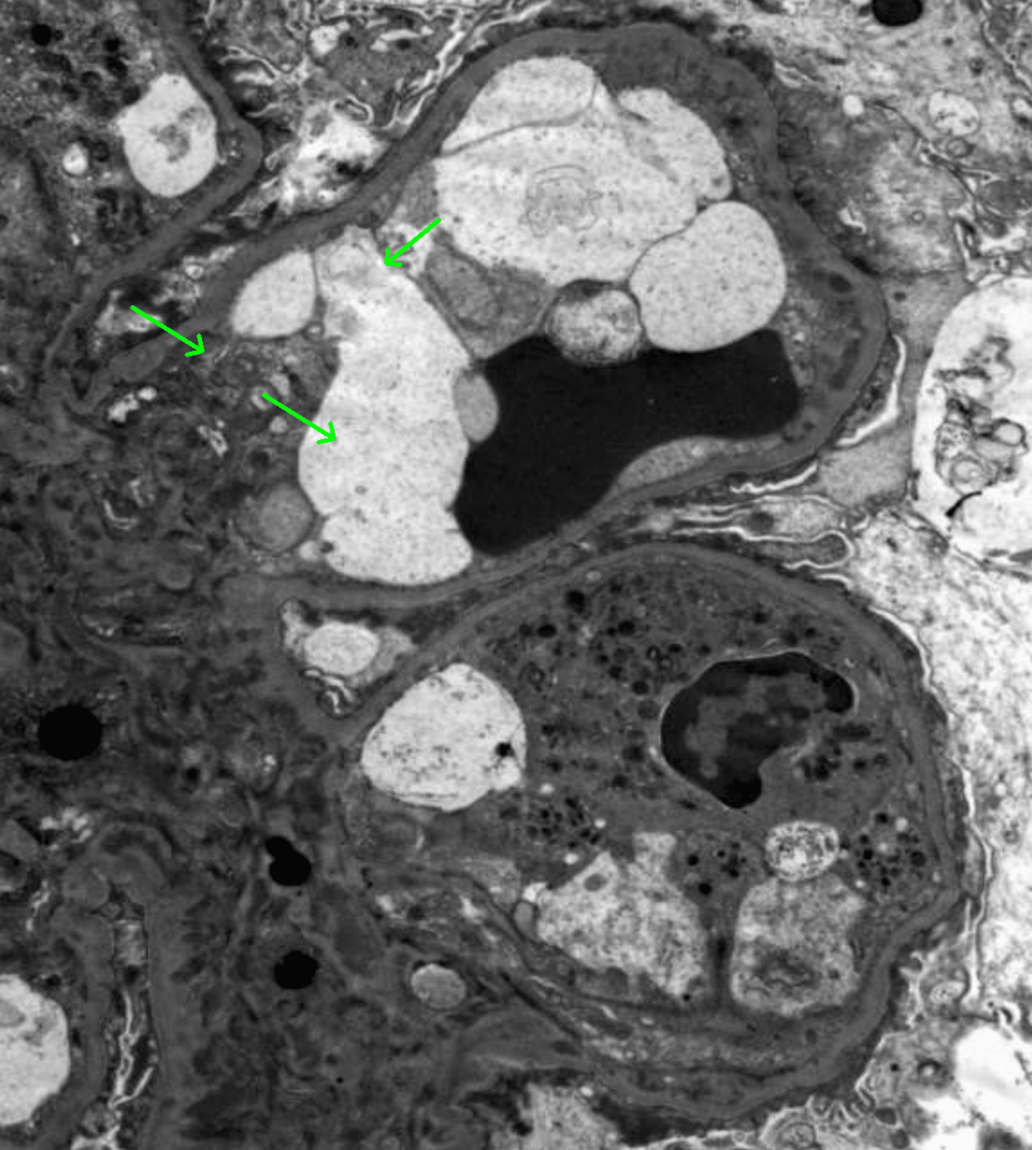
Electronic Microscopy (EM) shows frequent mesangial and scattered subendothelial immune complex deposits (green arrows).

A kidney biopsy was performed on hospital day two and histopathologic evaluation revealed focal endocapillary hypercellularity and segmental glomerular basement membrane thickening (Figures 2, 3). Immunofluorescence (Figure 4) demonstrated full-house positivity (IgG and light chains, weak for IgA, C3, and C1q, trace for IgM). Electron microscopy revealed subepithelial and mesangial electron-dense deposits (Figure 5). While IgA deposits may be present, as in IgA nephropathy, isolated or dominant IgA without C1q usually points away from lupus. Mixed immune-complex glomerulonephritis rarely involves all five markers, particularly C1q. Therefore, the presence of a full-house pattern supports lupus nephritis as the underlying diagnosis despite occasional IgA positivity. Based on the ISN/RPS 2003 classification system, the findings were diagnostic of Class III (focal proliferative).

Following biopsy, the patient was initiated on mycophenolate mofetil (MMF) in combination with steroid therapy. Hydroxychloroquine was also added to his regimen. The patient’s creatinine gradually improved over the next week, and he was discharged with close outpatient follow-up by nephrology, rheumatology and dermatology.

## Discussion

Systemic lupus erythematosus is a chronic autoimmune disease characterized by autoantibody production and immune complex deposition, leading to widespread tissue inflammation. Its clinical manifestation is heterogeneous, but renal involvement is a frequent and severe complication, affecting up to 60% of adults with SLE and serving as a major predictor of long-term morbidity and mortality [1,2]. Although LN typically presents within the first few years of SLE diagnosis, it may also be the initial manifestation, as in this patient who presented with rash, anemia, proteinuria, and AKI, ultimately diagnosed with SLE and a biopsy-proven LN.

This patient’s renal biopsy revealed features consistent with focal proliferative lupus nephritis, classified as Class III according to the 2004 ISN/RPS criteria. Class III LN is defined by active or chronic immune complex-mediated glomerulonephritis involving fewer than 50% of all glomeruli, with either segmental or global lesions. Typical histopathologic features include endocapillary hypercellularity, leukocyte infiltration, subendothelial immune complex deposition, and capillary loop necrosis. These findings may be accompanied by crescent formation and interstitial inflammation. Clinically, patients often present with hematuria, proteinuria, and mild to moderate renal dysfunction. Although the prognosis of Class III LN is generally better than that of diffuse proliferative LN (Class IV), it carries a significant risk of progression without appropriate immunosuppressive therapy [6].

Before the renal biopsy confirmed the diagnosis, several differential diagnoses were considered, given the patient’s presentation with rash, AKI, and systemic symptoms. One major consideration was antineutrophil cytoplasmic antibody (ANCA)-associated vasculitis, which often presents with rapidly progressive glomerulonephritis, constitutional symptoms, and skin manifestations such as palpable purpura. However, this patient lacked respiratory involvement (e.g., hemoptysis, sinus disease), had a negative ANCA panel, and exhibited serologic markers more characteristic of SLE, specifically, positive ANA, high anti-dsDNA titers, and hypocomplementemia [8].

Drug-induced acute interstitial nephritis (AIN) was also considered, as it can present with AKI and nonspecific systemic symptoms, sometimes accompanied by rash. However, there was no recent use of medications commonly associated with AIN, such as antibiotics, nonsteroidal anti-inflammatory drugs (NSAIDs), or proton pump inhibitors. Additionally, the presence of proteinuria, hematuria, and active urinary sediment made AIN less likely [9].

Another potential mimic was IgA nephropathy, particularly given the presence of hematuria and AKI. While IgA nephropathy can present acutely, especially in the setting of infection, it typically lacks systemic findings such as rash and anemia. Serologic studies in IgA nephropathy are usually negative, and complement levels remain normal, further differentiating it from SLE [10].

Despite the diagnostic uncertainty pending the biopsy, the combination of clinical features (photosensitive rash, anemia, proteinuria, and hematuria) along with a serologic profile of high ANA and anti-dsDNA levels and low complement (C3 and C4), created a strong pre-biopsy suspicion for lupus nephritis. In such scenarios, early initiation of immunosuppressive therapy is often justified to prevent irreversible renal damage while awaiting definitive histopathologic confirmation. Accurate classification of LN is essential, as treatment intensity and prognosis vary across classes. As class III LN can progress to more severe diffuse involvement (Class IV) if left untreated, prompt initiation of immunosuppressive therapy is therefore critical to prevent disease progression and preserve renal function. In this case, the patient was started on high-dose corticosteroids and MMF for induction therapy, in accordance with current guidelines for Class III LN [11]. 

MMF is an established first-line agent for induction therapy in Class III LN and is preferred over cyclophosphamide in many cases due to its favorable side effect profile and equivalent efficacy in non-severe proliferative disease [12]. Corticosteroids remain a cornerstone of treatment to rapidly reduce inflammation. Hydroxychloroquine was also included in this patient’s treatment, as it has been shown to reduce disease flares, improve survival, and preserve renal function in SLE patients [13].

This case also emphasizes the diagnostic complexity of SLE and LN. The initial presentation included anemia, proteinuria, and a photosensitive rash - features that are nonspecific but suggestive of systemic autoimmunity. The presence of positive ANA and anti-dsDNA antibodies, low complement levels, and histopathologic confirmation established the diagnosis according to the 2019 European Alliance of Associations for Rheumatology (EULAR)/American College of Rheumatology (ACR) criteria [14]. Timely nephrology consultation and kidney biopsy enabled accurate classification and appropriate treatment.

The lack of follow-up beyond hospital discharge limits conclusions about renal trajectory and long-term response to therapy in this patient, however, prognostically, patients with Class III LN generally have a more favorable renal outcome compared to those with Class IV. However, the disease course can still be variable, particularly if treatment is delayed. Early response to therapy, adherence to immunosuppressive regimens, and close clinical monitoring are key determinants of long-term renal outcomes. Prompt diagnosis and appropriate management are critical to prevent progression to more advanced classes and chronic kidney damage [7].

This case highlights the importance of maintaining a high index of suspicion for lupus nephritis in patients with unexplained AKI and systemic signs suggestive of autoimmunity. It also accentuates the role of renal biopsy in stratifying risk and guiding therapy in patients with complex or atypical presentations. Multidisciplinary management and patient education remain key components of care to improve outcomes and minimize treatment-related complications.

## Conclusions

Clinicians should maintain a high index of suspicion for SLE in patients presenting with unexplained dermatologic manifestations, renal impairment, and hematologic abnormalities (even in male patients and in the absence of a prior history of autoimmune disease). While SLE is more prevalent in women of childbearing age, its occurrence in males is often underrecognized, leading to potential delays in diagnosis and treatment. Such delays can contribute to disease progression and irreversible organ injury, particularly when lupus nephritis is present. This case illustrates the need for vigilance in evaluating atypical presentations and reinforces the importance of a thorough workup that includes targeted serological testing, immunologic assays, and, when indicated, histopathologic confirmation via renal biopsy. Early and accurate diagnosis facilitates timely initiation of immunosuppressive therapy, which can significantly alter disease trajectory. Furthermore, the complexity of SLE management, especially when multiple organ systems are involved, highlights the importance of multidisciplinary collaboration among dermatology, nephrology, rheumatology, and pathology teams. Coordinated care not only improves diagnostic accuracy but also optimizes long-term outcomes, reducing the risk of chronic kidney disease and other irreversible complications associated with lupus nephritis. This case serves as a reminder that SLE should remain a diagnostic consideration across genders and age groups, particularly when constellation patterns of symptoms suggest a systemic inflammatory process.
